# A novel organic chromo-fluorogenic optical sensor for detecting chromium ions

**DOI:** 10.1016/j.heliyon.2024.e37480

**Published:** 2024-09-07

**Authors:** Sayed M. Saleh, Reham Ali, Azizah Algreiby, Bayader Alfeneekh, Ibrahim A.I. Ali

**Affiliations:** a Department of Chemistry, College of Science, Qassim University, Buraidah, 51452, Saudi Arabia; b Chemistry Department, Faculty of Science, Suez Canal University, Ismailia, Egypt

**Keywords:** Transition metals, Chromium, Fluorescence, Coordination reactions, Water contamination, Environmental monitoring

## Abstract

Sensing trivalent chromium ion (Cr(III)) is widely applied in different areas, such as clinical analysis, marine, environmental monitoring, or even chemical industry applications. Cr(III) has a significant role in the physiological process of human life. It is classified as an essential micronutrient for living organisms. Herein, we developed and designed a novel optical Cr(III) ions sensor film. The investigated sensor has a relatively small dynamic range of 1.24 × 10^−3^ to 0.5 μM. We report a highly sensitive optical sensor film for Cr(III) ions based on diethyl 3,4-diaminothieno[2,3-b]thiophene-2,5-dicarboxylate (*3D*) probe. The optical characteristics of the chemical probe exhibit substantial emission at 460 nm under 354 nm excitation. Besides, the interaction of the Cr(III) ions with *3D* involves a complex formation with a 2:1 (metal: ligand) ratio, which is convoyed by the main peak enhancement that centered at 460 nm of 3D, and the main peak is red-shifted to 480 nm. The easily discernible fluorescence enhancement effect is a defining characteristic of the complexation reaction between the 3D probe and Cr(III). On the basis of the substantial fluorescence mechanism caused by the formation of a (Cr(III)-3D complex, which inhibits the photo-induced electron transfer (PET) process, the devised optical sensor was proposed. This film exhibits exceptional sensitivity and selectivity due to its notable fluorescence properties, stock shift of less than 106 nm, and detection capabilities at a significantly low detection limit of 0.37 × 10^−3^ μM. The detection procedure is executed by utilizing a physiological pH medium (pH = 7.4) with a relative standard deviation RSDr (1 %, n = 3). In addition, the 3D sensor demonstrates a high degree of affinity for Cr(III), as determined by the calculation of its binding constant to be 1.40 × 10^6^. We present an impressive optical sensor that is constructed upon a three-dimensional molecule.

## 1 Introduction

Chromium is one of the essential ions that appear as trace levels in living systems and the thirteenth richest metal ion in the earth's crust [1]. It is a hard metal with a high corrosion resistance level. As a result, adding Cr(III) to steel significantly strengthens it against corrosion and discoloration [2] and is mainly employed for chromium plating [3]. It is also used in several important industries [4,5]. This crucial ion is a vital nutrient contributing to numerous biochemical processes. These processes include metabolizing carbohydrates, lipids, proteins, and nucleic acids [6,7]. The ion accomplishes this by reactivating particular metabolic enzymes, preserving proteins and nucleic acids, and producing hemoglobin in red blood cells [8].

However, the effects of chromium (III) are not always beneficial. It has the potential to activate insulin, thereby reducing blood glucose levels. A chromium deficiency (III) has been linked to an increased risk of diabetes and heart disease [9]. On the other hand, elevated concentrations of chromium (III) can disrupt regular enzymatic processes and cellular structures [10]. Furthermore, chromium (III) is an environmental pollutant that has reached hazardous levels due to industrial and agricultural activities. It has been classified as a mutagenic and carcinogenic agent by the US Environmental Protection Agency USEPA [11]. The World Health Organization (WHO) has set the maximum permissible level of chromium in drinking water at 0.1 mg/L (1.9 μM) [12].

When it comes to detecting metal ions, fluorescent probes have emerged as a popular choice due to their ease of preparation, high sensitivity, excellent selectivity, and low cost [13,14]. These probes work by interacting with metal cations through a chromophore, with changes in fluorescence intensity indicating the sensing process [15]. However, developing a fluorescence-based turn-on metal-sensing mechanism is a significant challenge, primarily due to the paramagnetic fluorescence quenching properties of metals like chromium (III) [16]. The paramagnetic property of chromium (III) is well-known for its ability to induce fluorescence quenching. Yet, the lack of a suitable recognition moiety/ligand combination further complicates the detection of chromium (III) using fluorimetric methods [17].

Optical chemical sensors for detecting heavy metals are based on various principles, including fluorescence [[18], [19], [20], [21], [22]], colorimetry [23,24], and surface plasmon resonance [25,26]. The sensing element can comprise various substances, including organic pigments [27,28], nanoparticles [29,30], polymers [31], enzymes [32], antibodies [33], and DNA [34]. Organic molecules are most widely synthesized to achieve this purpose of chemical sensing. They can interact with one another to selectively identify analytes of interest in industry, ecology, and medicine.

Using different types of organic compounds as substantial organic fluorescence probes has attracted much interest recently. As a result of the nitrogen atom that exists in some organic compounds, as in the amine or imine groups serving as π-acceptor [35], they can play an essential part in coordination chemistry. They have been utilized as a detection tool in the analysis of a variety of metal ions. In addition, the number of coordinated atoms is frequently used to classify these compounds. They have a variety of advantageous properties, such as simple synthesis, a broad color scale for the compounds that are formed, stability in metal complexes, and a wide range of potential applications due to their high levels of thermal and mechanical strength. They have various uses, including analytical, bioanalytical chemistry, and material sciences. In addition, different types of organic molecules have emerged as an essential factor in elaborating new structures with a wide range of industrial and biological applications in many different chemical reactions. Another example is organic molecules comprising nitrogen, oxygen, and sulfur atoms that are antipyretic medications [36,37].

Numerous established methodologies are commonly employed for detecting Cr(III) ions in diverse samples. These techniques encompass atomic absorption spectroscopy (AAS) [38], inductively coupled plasma atomic emission spectroscopy (ICP-AES) [39], instrumental neutron activation analysis [40], electrochemical methods [41], X-ray fluorescence spectroscopy [42], electrothermal atomic absorption spectrometry (ETAAS) [43], and flame atomic absorption spectroscopy (FAAS) [44]. Nevertheless, the current methods employed for detection exhibit a high degree of technological intricacy and need the use of costly apparatus. Furthermore, these approaches could be more satisfactory regarding sensitivity since they show a lack of responsiveness at elevated concentration levels. Additionally, the sample preparation process associated with these methods is time-consuming [45]. The optical responses of the sensors are illustrated by a variety of mechanisms, such as chelation-enhanced fluorescence (CHEF) [46], intermolecular charge transfer (ICT) [47], fluorescence resonance energy transfer (FRET) [48], metal-to-ligand charge transfer (MLCT) [49], photoinduced electron transfer (PET) [50], excited state intermolecular proton transfer (ESIPT), aggregation-induced emissions (AIE) [51], and photoinduced charge transfer (PCT) [52].

This study presents a newly developed optical sensor film that utilizes a diethyl 3,4-diaminothieno[2,3-b]thiophene-2,5-dicarboxylate (*3D)* organic fluorophore to detect Cr(III) ions. The mechanism can be ascribed to metal-ligand chelation, where a highly sensitive *3D* probe and Cr(III) ions combine to create a metal complex. The metal complex's structure is accompanied by a significant increase in fluorescence owing to the creation of a metal: ligand complex, which prevents the PET process [53,54]. The detection mechanism of Cr(III) in the presence of *3D* has been analyzed by UV–Vis and fluorescence measurements. The new optical sensor exhibits exceptional selectivity, a substantial limit of detection (LOD), sensitivity, and rapid reversibility for detecting Cr(III) metal ions.

## 2 Materials and methods

Sigma-Aldrich was the purchase source for all the chemicals employed. Other chemical ingredients and solvents were of analytical quality and were administered in the same manner as they were received. Methanol was of HPLC grade. Before any experiments, every metal nitrate stock solution was newly made with bi-distilled water and utilized immediately.

### 2.1 Instruments

NMR spectra were obtained using a JEOL JNMECA 600 spectrometer capable of operating at 600-MHz for ^1^H and 150-MHz for ^13^C. The TMS was used as an internal standard. A Kofler Microhot Stage Instrument was utilized to determine melting points. An API QSTAR pulsar mass spectrometer was utilized to record the mass spectrum of the separated chemical. Using an Evolution™-200-series/UV–Visible spectrophotometer. In a quartz cell measuring 1 cm in diameter, fluorometric measurements were taken using a JASCO FP-6300 spectrofluorometric. These measurements included excitation and emission spectra.

### 2.2 Synthesis of 3D probe

**Scheme 1:**
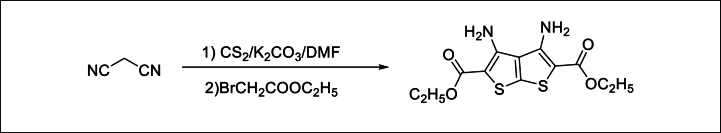
Synthesis of 3D compound.

A mixture of 0.5 mol (69.0 g) oven dried potassium carbonate (K_2_CO_3_) and 0.1 mol (6.6 g) of the malononitrile in 100 mL DMF was stirred at room temperature for 1 h, then the mixture was cooled to 0 °C and 0.1 mol (6.0 mL) carbon disulfide were added dropwise under vigorous stirring. After 30 min, the mixture was cooled to 0 °C again and 0.2 mol (22.0 mL) ethyl bromacetate was added in 20 min. The reaction mixture was then stirred for 5 h at 50 °C, 3 h at 90 °C and poured into 300 mL of cold water. The precipitate was collected and washed 3 times with 100 mL of water. The crude diethyl 3,4-diaminothieno[2,3-b]thiophene-2,5-dicarboxylate obtained were purified by crystallization in 83 % yield (see Scheme 1). ^1^H NMR (300 MHz, CDCl_3_) δ = 4.79 (s, 4H, 2NH_2_), 3.96 (q, J = 7.1 Hz, 4H, 2OCH_2_), 0.98 (t, J = 7.1 Hz, 6H, 2CH_3_) (Fig. S1). ^13^C NMR (75.0 MHz, CDCl_3_) δ = 161.2, 144.8, 136.8, 127.0 (C-Ar), 121.4 (Olefinic), 61.2 (2OCH_2_), 14.0 (2CH_3_). Ms data; *m*/*z*: 314(100 %), 258, 222, 170, 166 (Fig. S2) [55].

### 2.3 Fabrication of 3D film

**Scheme 2:**
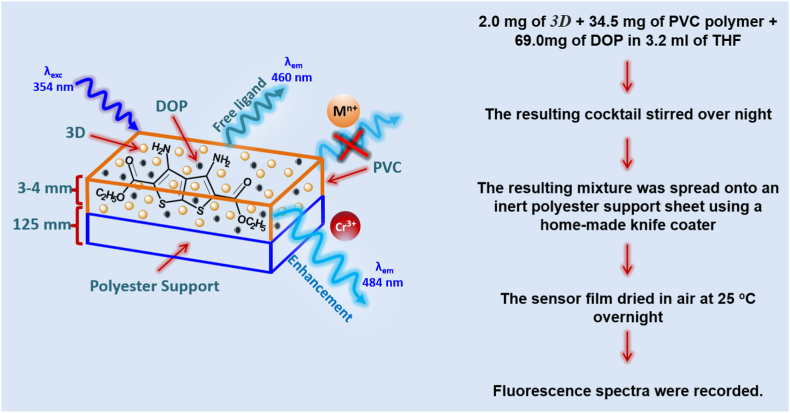
3D optical sensor film for Cr(III)ions.

Mixing 2.0 mg of *3D*, 34.5 mg of polyvinyl chloride PVC, and 69.0 mg of bis(2-ethylhexyl) phthalate (DOP) plasticizer in 3.2 mL of tetrahydrofuran (THF) resulted in the chemical sensor film for fabrication. After that, for around 8 h, the net solution was agitated until it became transparent. A polyester polymer support was used to disseminate the resulting cocktail using a knife coater [56]. Air was allowed to dry the optical sensor film. Based on the materials composition, it was determined that the sensor layer had a thickness of 3–4 μm as shown in Scheme 2.

### 2.4 Optical measurements

An experiment series was carried out to investigate the *3D* chromophore optical characteristics. Experiments were conducted with a concentration of 0.5 μM 3D in a buffered medium based on H_2_O:ethanol (5:95) and a constant pH of 7.4 to measure UV–Vis absorbance. The buffered medium contained 20 mM HEPES. The Cr(III) concentration range was between 0 and 130 nM. At 25 °C, the net volumes of the solutions collected from the *3D* chemical probe and the Cr(III) aliquots were maintained at a constant 2 mL throughout the titration phases. The fluorimetric studies for the *3D* probe were carried out in the presence of Cr(III) under the identical circumstances studied. In addition, the optical characteristics of the *3D* probe were investigated in the presence of 0–1.1 μM Cr(III) to assess the chemical sensor's effectiveness.

### 2.5 Binding study

We used Job's approach [5,7], [57]. [58] to measure the stoichiometry of the *3D* chromophore and Cr(III) ions. At a buffer solution with a pH of 7.4, the complexation process was conducted by combining Cr(III) metal ions with a 0.5 μM *3D* chemical probe at equal molar concentrations. From 9:1 to 9:1, the molar ratios of the reactants were changed. At 480 nm, the *3D* probe exhibited its greatest fluorescence.

## 3 Results and discussion

### 3.1 Optical characterization of the 3D probe

**Fig. 1:**
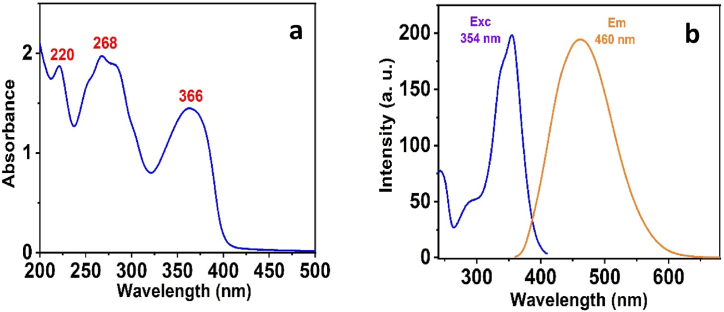
(a) Absorbance spectrum of 3D, (b) Fluorescence spectrum of 3D (*λ*_em_ 460; *λ*_exc_ 354 nm).

The chemical sensor displays unique optical properties in its prepared state. UV–Vis spectroscopy was measured. The UV–vis spectrum of ligand displayed a characteristic absorption peak at 220, 268, and 366 nm correspondingly (see Fig. 1a). In addition, the *3D* chromophore exhibits an apparent peak at 460 nm under excitation with 354 nm (see Fig. 1b).

### 3.2 Sensing measurements

**Fig. 2:**
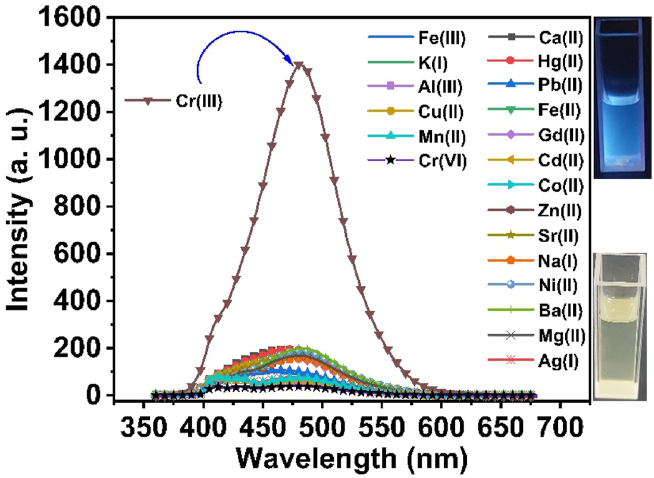
Fluorescence spectra of 3D probe in the presence of different metal ions.

The fluorescence emission intensity of *3D* was measured at a wavelength of 460 nm upon excitation at 354 nm; it was observed that the 3D molecule has a weak fluorescence. This can be attributed to the photoinduced electron transfer (PET) process, where the lone pair of electrons of the N atom on the amine group, which acts as an electron donor, transfer to the adjacent fluorophore (thiophene ring), which acts as an acceptor; as a result, a weak fluorescence is obtained. When Cr(III) is added to the *3D* probe, it exhibits a significant increase in fluorescence because it forms a 1:2 complex (*3D*-Cr(III)) where the amino group acts as a receptor for the Cr(III) ions, and the formation of Cr(III)-*3D* complex prevents the PET process, resulting in the fluorescence enhancement of *3D* probe [53,54]. Alkali, alkaline, and other transition metal ions did not impact the fluorescence. This suggests that the *3D* compound is a highly specific Cr(III) fluorescent sensor. Cr(III) ion was introduced into a combination including *3D* and other potentially competitive metal ions indicated above. The mixture was prepared using a buffered medium of H_2_O: C_2_H_5_OH (5:95) and maintained at a constant pH of 7.4 using a 20 mM HEPES solution. This increased fluorescence intensity, as seen in Fig. 2. These data indicate that *3D* can potentially serve as a highly sensitive and specific fluorescence optical sensor for Cr(III) ions.

**Fig. 3:**
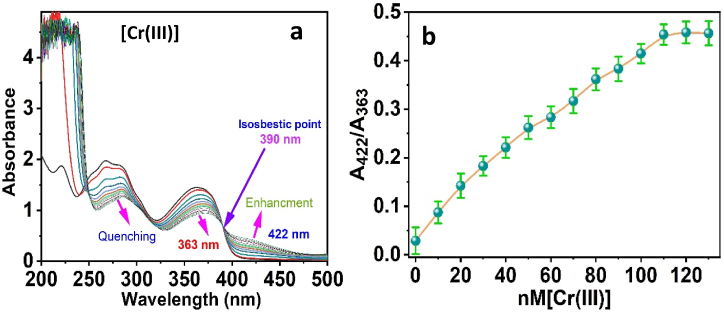
(a) The absorbance spectra of 3D with the addition of Cr(III) ions; (b) The absorbance band ratios (A_422_/A_363_) versus Cr(III) ion concentrations.

The reaction between ligand and chromium ions was analyzed using absorbance and fluorescence techniques. *3D* ligand's UV–vis spectrum displayed characteristic absorption peaks, which possibly will be attributed to π-π* and n-π* transitions [59]. With the gradual addition of Cr(III) ions to the *3D*, we note an enhancement in the peak, and the absorbance was at 422 nm, with an isosbestic point at 390 nm. The intensity of the spectrum displayed crucial quenching at (363 nm) (Fig. 3a). It is interesting to note that the reaction of the *3D* chromophore UV–vis spectra confirms the binding process comprising the Cr(III) ions and the functional groups, which contain oxygen and nitrogen atoms of the *3D* [60]. Notably, there is a correlation between the absorption ratio of the *3D* probe at 422 and 363 nm and the concentration of Cr(III) across the dynamic range of 0–130 nM (Fig. 3b). In particular, when the Cr(III) concentration rises to a molar ratio of ligand to Cr(III) of 1:2, the absorption ratios become constant at higher molar ratios. Consequently, this could be attributed to encapsulating the Cr(III) ions via coordination with the chemical ligand's oxygen and nitrogen donor sites [61].

**Fig. 4:**
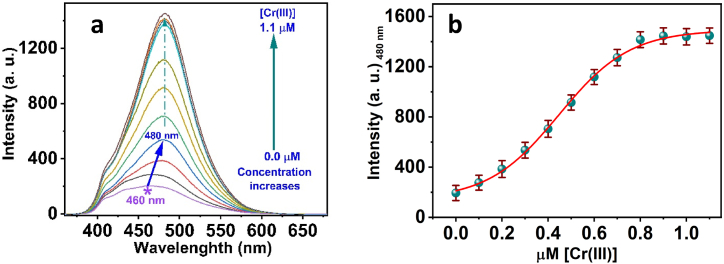
(a) The fluorescence emission spectra of *3D* in the presence of various [Cr(III)]; (b) the Fluorescence spectra of *3D* versus molar concentration of Cr(III) ions.

The Cr(III) was detected by the ligand in the fluorescence spectra with the gradual addition of Cr(III) concentration, as shown in (Fig. 4a). A pH 7.4 buffer system tuned to 20 mM HEPES was used to study the fluorimetric titration response between the chemical probe *3D* and concentrations of Cr(III). When excited at 354 nm, the *3D* chemical probe shows a maximal fluorescence band at 460 nm (Fig. 4a). The addition of Cr(III) ions to the buffered solution at a molar ratio of 7.9 × 10^−3^ to 1.1 μM Cr(III) ions immediately increased the emission of the major *3D* peak at 460 nm. The appearance of a strong fluorescence peak at 480 nm with a 20 nm red shift from the *3D* main peak proves the formation of Cr(III)- *3D* complex. An increase in the concentration of Cr(III) metal ions, with a dynamic range of 7.9 × 10^−3^ to 1.1 μM, significantly brightened the fluorescence band at 480 nm, providing further proof that the *3D* chemical probe had complexed with Cr(III) ions. This suggests that the Cr(III)-*3D* chelation has an equivalent ratio of 2:1 Cr(III):*3D*, as the *3D* fluorescence remained unchanged upon repeated addition of Cr(III) at a molar concentration of >2 equivalent (Fig. 4b).

**Fig. 5:**
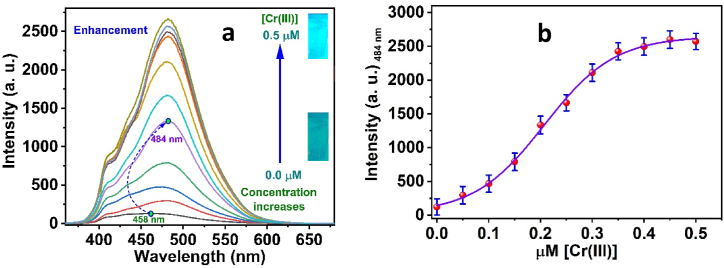
(a) The fluorescence spectra of the *3D* sensor film in the presence of various Cr(III) ion concentrations; (b) the fluorescence intensities of *3D* sensor film versus molar concentration of Cr(III) ions.

Fig. 5a shows the results of determining the *3D* sensing film's fluorescence spectra in the presence of different Cr(III) ions concentrations. A significant increase in the emission band at 458 nm, accompanied by a 26 nm red shift (to 484 nm), was noted during the fluorescence titration. An increase in the primary emission peak's fluorescence intensity at 458 nm with a red shift at 484 nm was evidence of complex development. For confirmation of the production of the Cr(III)-*3D* complex, Fig. 5b shows that (F_484_) is dependent on [Cr(III)] through correlation. One possible explanation for the complexation process is that *3D* acts as a donor chelator and binds to Cr(III) as a receptor. Amazing sensitivity to Cr(III) was shown in the *3D* probe molecule throughout a concentration range of 1.24 × 10^−3^ to 0.5 μM. The LOD was determined using the 3D fluorimetric titration with Cr(III). Assuming an accuracy of ±1 % in perceiving fluorescence intensity, the LOD is predicted to be 0.37 × 10^−3^ μM Cr(III). Therefore, the 3D chemical probe is an optical chemosensor that detects Cr(III) with great impact. Memorably, the response time of the optical sensor film was determined to be 1.18 min.

### 3.3 PH effect on the sensing process and time response

**Fig. 6:**
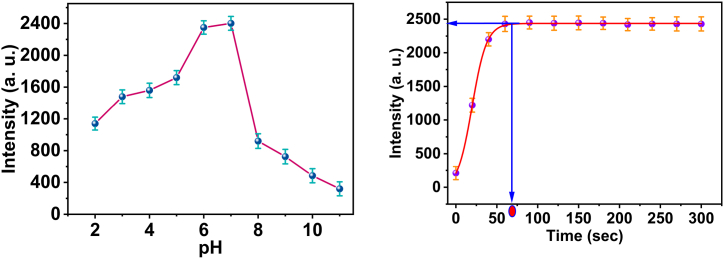
(a) The alteration in fluorescence intensity with the pH of the sensors *3D* with Cr^3+^; (b) the response time of the optical sensor to Cr(III) ions.

Most metal cations are detected by influencing the fluorescence of an optical sensor, which is associated with hydrogen proton transfer. Variations in the pH of the surrounding solution impact this phenomenon. The study investigated how pH levels ranging from 2 to 11 affect the selectivity and responsiveness of the chemical sensor towards Cr(III) ions. By manipulating the pH of the liquid solution using 20 M HEPES buffer solutions at a specific concentration of Cr(III) ions (0.5 μM) and stimulating it with light at a wavelength of 354 nm, we successfully measured the concentration of Cr(III) ions (as shown in Fig. 6a). Within the pH range of 2.0–7.0, the response of the chemical sensor is directly proportional to the pH value. This is because the 3D probe remains protonated without forming any complexes. The proton's attachment to the nitrogen and oxygen atoms of the active groups hinders the creation of complexes to some extent. The observed elevation in 3D sensor fluorescence when exposed to alkaline pH or pH levels over 7 results from forming Cr(III) hydroxide from the liquid solution. The study examined the reactivity and selectivity of the 3D sensing molecule for Cr(III) ions in various pH 7.4 buffered systems, such as the HEBES buffer.

In order to measure the response time of the sensor film to Cr(III) ions, a fluorimetric technique was employed to detect the luminescence intensity of the optical sensor subsequent to immersed the sensor film in 0.5 μM Cr(III). The primary peak fluorescence intensity of the *3D* probe exhibited a gradual increase until it reached a constant value approximately 1.08 min. There is no alteration observed in the blue fluorescence of *3D* upon the introduction of Cr (III) (See Fig. 6b). The stability of the chemosensor was assessed by submerging it in an aqueous solution for a duration of 12 h. During this time, neither the emission of *3D* fluorescence nor the leaching of the *3D* probe content were observed.

### 3.4 Binding efficiency

**Fig. 7:**
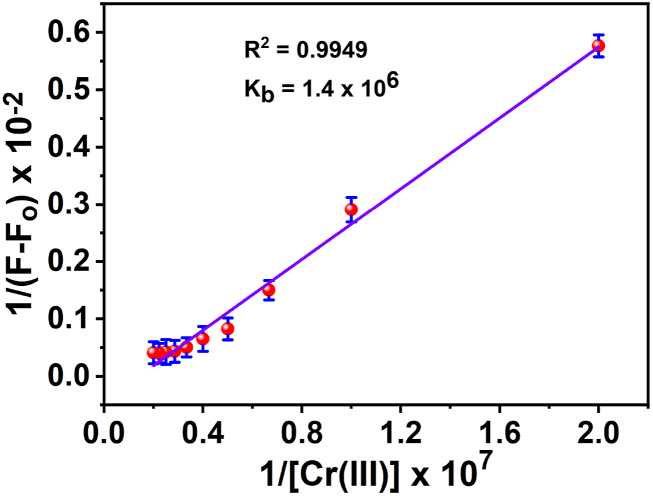
Benesi-Hildebrand plot for the Cr(III)-3D complex.

Based on the Benesi-Hildebrand equation and the plotting 1/(F_o_-F) against 1/[Cr(III)] in (Fig. 7), the binding constant (K_b_) for *3D* was estimated to be 1.4 × 10^6^.

Benesi-Hildebrand equation:

Where F_o_ is the fluorescence intensity of the ligand in the absence of the Cr(III), and F is the fluorescence intensity in the presence of the Cr(III). K is the binding constant, and F_max_ is the fluorescence intensity in the presence of added [M]_max_ (max. metal concentration). The slope of the linear graph of 1/(F-F_o_) against 1/[M]^n^ allowed for the determination of the association constant (K_a_). More importantly, the plot was linear (R^2^ = 0.9949) in this range, submitting that *3D* ligand can be employed to estimate Cr(III) concentration. The affinity of the 3D probe for Cr(III) directly affects the sensitivity of the corresponding sensor. The Cr(III)-3D complex has a high affinity for binding, suggesting that *3D* will possess greater sensitivity. Even low concentrations of the ligand will trigger a response. Conversely, the sensor sensitivity will be reduced if the chemosensor has low binding efficiency. Thus, Higher ligand concentrations will be required to elicit a response.

### 3.5 Interference investigation

**Fig. 8:**
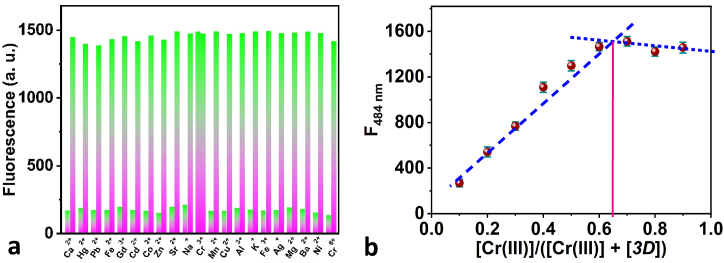
(a) the selectivity of 3D ligand toward other competing ions in the absence or the presence of Cr(III) ions; (b) Job's plot for the stoichiometry detection of the Cr(III)-*3D* complex.

In interference studies, has been added of various metal ions at a concentration of 0.5 μM. However, in the presence of Cr(III), the sensor film exhibits enhancement of fluorescence emission intensity without any interference from other competitive metal ions present in the solution, as evident in (Fig. 8a). Therefore, the sensor film has the potential to function as a very precise sensor for Cr(III). Herein, our findings indicate that Cr(III) can greatly increase the fluorescence intensity of the 3D probe. This allows us to effectively detect and estimate even small amounts of Cr(III) in a water-based solution. However, we have observed that Cr(VI) does not impact the fluorescence intensity of the 3D probe. Therefore, to detect Cr(VI), we have to convert Cr(VI) into Cr(III) by utilizing suitable reducing agents [62]. For the determination of stoichiometry between ligand and Cr(III), Job's plot analyses [63] were applied (Fig. 8b). The Job's plot was produced by altering the [Cr(III)]. A maximum fluorescence was when the molar fraction of Cr(III) reached approximately 0.65, indicating that the stoichiometric binding ratio among Cr(III) ions and *3D* ligand is 2:1.

### 3.6 Reversibility

**Fig. 9:**
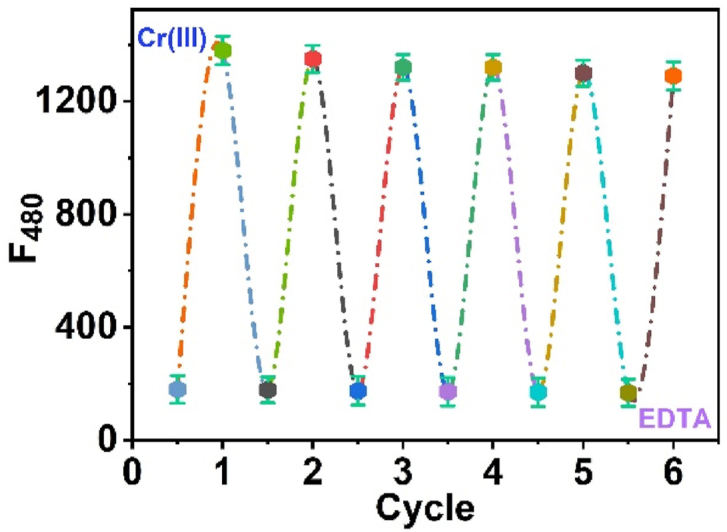
Reversibility of *3D*-Cr(III) and EDTA mutually, *λ*_ex_ = 354 nm and *λ*_em_ = 384 nm, respectively.

Reversibility is a very significant feature of an optical sensor. Ethylenediaminetetraacetate (EDTA), an extremely potent chelating agent for Cr(III) ions, was utilized to examine the sensor reversibility. EDTA solution considerably enhances the luminescence of the Cr(III)-*3D* complex because Cr(III) ions interact with the active groups of EDTA molecules. The emission intensity of the optical sensor was enhanced in the presence of Cr(III); upon the addition of EDTA, an immediate Cr-EDTA complex formed. Intriguingly, the fluorescence of the *3D* chemical probe decreases after the exchange chelation process. We can observe that the emission intensity of the *3D* quenched with EDTA addition and reached its original maximum after coordinating to the EDTA with 1:1 metal/*3D* ligand; the luminescence of ligand after six cycles reaches ≈92 % from its starting fluorescence as seen in (Fig. 9).

### 3.7 Quantum yield

Fluorescence quantum yields (QY) were calculated using quinine sulfate as a standard and reference; in sulfuric acid, a solution of quinine sulfate with a QY of 55 % was established. Using

the following equation, the fluorescence QY of the ligand was calculated [64].

$$Q_{X}=Q_{R\;}\frac{A_{R}{\;I}_{s}n_{s}^{2}}{A_{X}{\;I}_{R}{\;n}_{s}^{2}}$$

While.

• X and R point to the ligand and reference solutions
• η is a refractive index at room temperature
• I the integrated area under the peak
• A is the maximum absorbance peak.

Both the free (*3D*) and Cr(III)-*3D* complexes were found to have estimated quantum yield values of 0.173 and 0.285, respectively.

### 3.8 Assessment of Cr(III) in real samples

To validate the suggested approach, the synthesized probe 3D was utilized to quantify the concentration of Cr(III) in drinking and tap water samples. Because the examined samples did not contain any Cr(III), it may be concluded that this metal ion was not present. The recovery investigations were performed on additional water samples tainted with known concentrations of Cr(III). Each spike sample was subjected to three separate tests; the results of these findings are shown in Table 1. The additional data and the estimated values were found to be in good agreement with one another, as can be seen in the table, which demonstrates that our suggested technique is accurate. It is clear from the low relative standard deviation (RSD) percentage and the high recoveries that 3D has the potential to be used as a sensor for correctly identifying Cr(III) in actual samples. The obtained results were compared to the ICP-MS data, indicating that this method effectively detects Cr(III) in the analyzed materials.

**Table 1:** Detection of Cr(III) in various water.

Sample	added (μM)	ICP-MS (μM)	Found (μM)	RSD (%,n = 3)	Recovery (%)
Tap Water	0	0.00002	0	2.95	–
	0.05	0.0502	0.053	2.35	106.0
	0.2	0.201	0.211	2.19	105.5
	0.4	0.402	0.435	2.08	108.8
Mineral Water	0	0.00001	0	2.63	–
	0.05	0.0501	0.052	2.21	104.0
	0.2	0.203	0.207	2.12	103.5
	0.4	0.402	0.411	2.02	102.8

### 3.9 Comparison

The 3D chemosensor designed for fluorescence detection of Cr^3+^ is quite impressive. Furthermore, we conducted a comparison between the current sensor and previously documented chemosensors for Cr^3+^ as shown in Table 2. Chemosensor 3D demonstrated a comparatively superior response.

**Table 2:** Comparative analysis of chemosensor 3D with previously reported sensors.

Chemosensor	Analytes	Method	Mechanism	Medium	LOD (M)	Ref.
Carbazole-based Schiff base (1-(5-(9-hexyl-9H-carba-zol-3-yl)thiophen-2-yl)-N-(3-nitrophenyl) methanimine	Fe^3+^ and Cr^3+^	Turn-on Fluorescence	C=N isomerization inhibits ESIPT	CH_3_CN	2.75 × 10^−6^	[65]
A Schiff base based on triphenylamine and thiophene	Cr^3+^	Turn- off-on Fluorescence	PET	THF/H_2_O (1:1, v:v)	1.5 × 10^−6^	[66]
A thiophene-coumarin hybrid molecule	Cr^3+^	Turn-on Fluorescence	Complex formation inhibits PET	CH_3_CN-HEPES (0.02 M) (4:6, v/v)	1 × 10^−6^	[67]
Imine-linked, benzimida-zole-based chemosensor	Mg^2+^ and Cr^3+^	Turn-on Fluorescence	Cr^3+^ complex enhances Keto-tautomer	CH_3_CN/H_2_O (8:2, v/v)	7.94 × 10^−5^	[68]
2,3-dimethyl-4-(3-oxo-1,3-diphenylpropylidene-amino)-1-phenyl-1,2- dih-ydropyrazol-5-one	Al^3+^ and Cr^3+^	Turn-off-on Fluorescence	PET	CH_3_OH/H_2_O (8:2, v/v)	0.73	[69]
2-((quinolin-8-ylimino) methyl) naph-thalen-1-ol	Al^3+^ and Cr^3+^	Colorimetric	Complex formation Inhibits C=N isomerization	CH_3_OH	1.1 × 10−5	[70]
N,N0-bis(salicylidene)-2-(6-(2-aminophenyl)-4-phenylpyridin-2-yl)	Cr^3+^	Turn-on Fluorescence	PET and CHEF	CH_3_CN/H_2_O (95/5 %)	2.2 × 10^−7^	[71]
Diethyl 3,4-diaminothie-no[2,3-b]thiophene-2,5-dicarboxylate	Cr^3+^	Turn-on Fluorescence	Complex formation inhibits PET	H_2_O: C_2_H_5_OH (5:95) 20 mM HEPES	0.37 × 10^−9^	This work

## 4 Conclusion

In this study, we have created a novel optical sensor film using diethyl 3,4-diaminothieno[2,3-b]thiophene-2,5-dicarboxylate (*3D*) to detect Cr(III). The *3D* molecules exhibited preferential recognition of Cr(III) ions compared to other metal cations. Unlike the typical phenomenon of metal-induced fluorescence amplification, the presence of Cr(III) ions resulted in a notable increase in the fluorescence of the ligand mentioned. The optical film properties of the *3D* molecules were studied in the presence of Cr(III) in a combination of H_2_O: ethanol (5:95) based buffered medium with a constant pH of 7.4 using (20 mM HEPES). The investigation focused on the dependence of the film features on fluorescence. The *3D* optical sensor film has excellent selectivity and sensitivity towards Cr(III) ions, with a low limit of detection (LOD) of 0.37 × 10^−3^ μM. It does not show any interference from neighboring cations in the medium. The ligand's complexation with Cr(III) ions demonstrated reversibility when treated with EDTA, allowing the regeneration of the free ligand for further sensing of Cr(III). Based on the plot of Job's analysis, the binding stoichiometry between Cr(III) and the ligand, as determined by the fluorescence approach, was found to be 2:1. The Benesi-Hildebrand equation was employed to determine the binding constant (K_b_) of Cr(III) to the ligand, yielding a value of 1.4 × 10^6^. This optical sensor can be a notable tool for detecting Cr(III) in real water samples.

## Data availability statement

Data is included in the article/supp. material/referenced in the article.

## CRediT authorship contribution statement

**Sayed M. Saleh:** Writing – review & editing, Writing – original draft, Validation, Supervision, Methodology, Data curation, Conceptualization. **Reham Ali:** Writing – original draft, Supervision, Methodology, Conceptualization. **Azizah Algreiby:** Validation, Investigation. **Bayader Alfeneekh:** Visualization, Validation, Methodology, Data curation. **Ibrahim A.I. Ali:** Visualization, Validation, Methodology.

## Declaration of competing interest

The authors declare the following financial interests/personal relationships which may be considered as potential competing interests: Sayed M. Saleh reports financial support was provided by 10.13039/501100007414Qassim University, 10.13039/501100023674Deanship of Scientific Research. If there are other authors, they declare that they have no known competing financial interests or personal relationships that could have appeared to influence the work reported in this paper.

## References

[1] Maity M.B., Talukdar D., Dutta B., Bairy G., Murmu N., Das G., Sinha C. (2023). Application of a Rhodamine-chromone Schiff base probe for sensing Fe^3+^, Al^3+^, and Cr^3+^ at low concentration and exploring the anticancer activity and bio-imaging. Inorg. Chim. Acta..

[2] Kamali S., Arabahmadi R., Orojloo M., Amani S. (2023). A new azo Schiff base probe for detection of Cr^3+^, HSO4^−^, and CN^−^: computational studies, 4-to-2 encoder, and integrated molecular logic circuits. Microchem. J..

[3] Singh J., Kaur V., Singh R., Bhardwaj V.K. (2018). Exploration of solvent responsive Cr3+-Schiff base conjugates formonitoring Cr^3+^ ions and organophosphates: fabrication of spot-testingdevices. Spectrochim. Acta Mol. Biomol. Spectrosc..

[4] Jebnouni A., Teka S., Bahrouni Y., Jaballah N.S., Bechambi O., Majdoub M. (2022). Highly selective turn-on fluorescent chemosensor for the detection of Cr(III) ion in drinking water. Opt. Mater..

[5] Musikavanhu B., Zhang Y., Zhu D., Xue Z., Yuan R., Wang S., Zhao L. (2022). Turn-off detection of Cr (III) with chelation enhanced fluorescence quenching effect by a naphthyl hydrazone Shiff base chemosensor. Spectrochim. Acta Mol. Biomol. Spectrosc..

[6] Wang M., Wang J., Xue W., Wu A. (2013). A benzimidazole-based ratiometric fluorescent sensor for Cr^3+^ and Fe^3+^ in aqueous solution. Dyes Pigments.

[7] Chalmardi G.B., Tajbakhsh M., Hasani N., Bekhradnia A. (2018). A new Schiff-base as fluorescent chemosensor for selective detection of Cr^3+^: an experimental and theoretical study. Tetrahedron.

[8] Sunnapu O., Kotla N.G., Maddiboyina B., Asthana G.S., Shanmugapriya J., Sekar K., Singaravadivel S., Sivaraman G. (2017). Rhodamine based effective chemosensor for Chromium (III) and their application in live cell imaging. Sensor. Actuator. B Chem..

[9] Lee S.Y., Bok K.H., Kim J.A., Kim S.Y., Kim C. (2016). Simultaneous detection of Cu^2+^ and Cr^3+^ by a simple Schiff-base colorimetric chemosensor bearing NBD (7-nitrobenzo-2-oxa-1, 3-diazolyl) and julolidine moieties. Tetrahedron.

[10] Amitha G.S., Rajan V.K., Amritha B., Muraleedharan K., Vasudevan S. (2019). Betti base and its modified phthalonitrile derivative for the turn on fluorimetric detection of Hg2+ and Cr^3+^ ions. J. Photochem. Photobiol. Chem..

[11] Shrivastava R., Upreti R.K., Seth P.K., Chaturvedi U.C. (2002). Effects of chromium on the immune system. FEMS Immunol. Med. Microbiol..

[12] EFSA Panel on Contaminants in the Food Chain (CONTAM) (2015). Scientific Opinion on the risks to public health related to the presence of nickel in food and drinking water. EFSA J..

[13] Zhang M., Gong L., Sun C., Li W., Chang Z., Qi D. (2019). A new fluorescent-colorimetric chemosensor based on a Schiff base for detecting Cr^3+^, Cu^2+^, Fe^3+^ and Al^3+^ ions. Spectrochim. Acta Mol. Biomol. Spectrosc..

[14] dos Santos Carlos F., Nunes M.C., De Boni L., Machado G.S., Nunes F.S. (2017). A novel fluorene-derivative Schiff-base fluorescent sensor for copper(II) in organic media. J. Photochem. Photobiol. Chem..

[15] Baslak C., Kursunlu A.N. (2018). A naked-eye fluorescent sensor for copper (II) ions based on a naphthalene conjugate Bodipy dye. Photochem. Photobiol. Sci..

[16] Das B., Ghosh A., Dorairaj D.P., Dolai M., Karvembu R., Mabhai S., Im H., Dey S., Jana A., Misra A. (2022). Multiple ion (Al^3+^, Cr^3+^, Fe^3+^, and Cu^2+^) sensing using a cell-compatible rhodamine-phenolphthalein-derived Schiff-base probe. J. Mol. Liq..

[17] Yang Y., Xue H., Chen L., Sheng R., Li X., Li K. (2013). Colorimetric and highly selective fluorescence" turn‐on" detection of Cr^3+^ by using a simple schiff base sensor. Chin. J. Chem..

[18] Ali R., Ghannay S., Messaoudi S., Alminderej F.M., Aouadi K., Saleh S.M. (2022). A reversible optical sensor film for mercury ions discrimination based on isoxazolidine derivative and exhibiting pH sensing. Biosensors.

[19] Saleh S.M., El-Sayed W.A., El-Manawaty M.A., Gassoumi M., Ali R. (2022). Microwave-assisted rapid synthesis of luminescent tryptophan-stabilized silver nanoclusters for ultra-sensitive detection of Fe(III), and their application in a test strip. Biosensors.

[20] Saleh S.M., Almotiri M.K., Ali R. (2022). Green synthesis of highly luminescent gold nanoclusters and their application in sensing Cu(II) and Hg(II). J. Photochem. Photobiol. Chem..

[21] Saleh S.M., El-Sayed W.A., El-Manawaty M.A., Gassoumi M., Ali R. (2022). An eco-friendly synthetic approach for copper nanoclusters and their potential in lead ions sensing and biological applications. Biosensors.

[22] Ali R., Alfeneekh B., Chigurupati S., Saleh S.M. (2022). Green synthesis of pregabalin‐stabilized gold nanoclusters and their applications in sensing and drug release. Arch. Pharmazie.

[23] Ali R., Ali I.A., Messaoudi S., Alminderej F.M., Saleh S.M. (2021). An effective optical chemosensor film for selective detection of mercury ions. J. Mol. Liq..

[24] Saleh S.M., Ali R., Hegazy M.E.F., Alminderej F.M., Mohamed T.A. (2020). The natural compound chrysosplenol-D is a novel, ultrasensitive optical sensor for detection of Cu(II). J. Mol. Liq..

[25] Dhara P., Kumar R., Binetti L., Nguyen H.T., Alwis L.S., Sun T., Grattan K.T. (2019). Optical fiber-based heavy metal detection using the localized surface plasmon resonance technique. IEEE Sensor. J..

[26] Bakhshpour M., Denizli A. (2020). Highly sensitive detection of Cd(II) ions using ion-imprinted surface plasmon resonance sensors. Microchem. J..

[27] Ali R., Elshaarawy R.F., Saleh S.M. (2017). Turn-on ratiometric fluorescence sensor film for ammonia based on salicylaldehyde-ionic liquid. J. Environ. Chem. Eng..

[28] Saleh S.M., Ali R., Ali I.A. (2017). A novel, highly sensitive, selective, reversible and turn-on chemi-sensor based on Schiff base for rapid detection of Cu(II). Spectrochim. Acta Mol. Biomol. Spectrosc..

[29] Ali R., Saleh S.M., Elshaarawy R.F. (2016). Turn-on pH nano-fluorosensor based on imidazolium salicylaldehyde ionic liquid-labeled silica nanoparticles. RSC advances.

[30] Saleh S.M., Ali R., Wolfbeis O.S. (2011). Quenching of the luminescence of upconverting luminescent nanoparticles by heavy metal ions. Chem.--Eur. J..

[31] Skorjanc T., Shetty D., Valant M. (2021). Covalent organic polymers and frameworks for fluorescence-based sensors. ACS Sens..

[32] Unnikrishnan B., Lien C.W., Chu H.W., Huang C.C. (2021). A review on metal nanozyme-based sensing of heavy metal ions: challenges and future perspectives. J. Hazard Mater..

[33] Wang Y., Zhang C., Liu F. (2020). Antibody developments for metal ions and their applications. Food Agric. Immunol..

[34] He Z., Yin H., Chang C.C., Wang G., Liang X. (2020). Interfacing DNA with gold nanoparticles for heavy metal detection. Biosensors.

[35] Yeldir E.K., Erdener D., Kaya İ. (2022). Synthesis and characterization of a pyrene-based Schiff base and its oligomer: investigation of fluorescent Cr^3+^ probe. React. Funct. Polym..

[36] Aroua L.M., Ali R., Albadri A.E., Messaoudi S., Alminderej F.M., Saleh S.M. (2023). A new, extremely sensitive, turn-off optical sensor utilizing schiff base for fast detection of Cu (II). Biosensors.

[37] Patil D.Y., Patil A.A., Khadke N.B., Borhade A.V. (2019). Highly selective and sensitive colorimetric probe for Al^3+^ and Fe^3+^ metal ions based on 2-aminoquinolin-3-yl phenyl hydrazone Schiff base. Inorg. Chim. Acta..

[38] Zhong W.S., Ren T., Zhao L.J. (2016). Determination of Pb (Lead), Cd (Cadmium), Cr (Chromium), Cu (Copper), and Ni (Nickel) in Chinese tea with high-resolution continuum source graphite furnace atomic absorption spectrometry. J. Food Drug Anal..

[39] Zhang L., Li Z., Du X., Li R., Chang X. (2012). Simultaneous separation and preconcentration of Cr (III), Cu (II), Cd (II) and Pb (II) from environmental samples prior to inductively coupled plasma optical emission spectrometric determination. Spectrochim. Acta Mol. Biomol. Spectrosc..

[40] Kumar R., Alamelu D., Acharya R., Rai A.K. (2014). Determination of concentrations of chromium and other elements in soil and plant samples from leather tanning area by Instrumental Neutron Activation Analysis. J. Radioanal. Nucl. Chem..

[41] Jin W., Wu G., Chen A. (2014). Sensitive and selective electrochemical detection of chromium (VI) based on gold nanoparticle-decorated titania nanotube arrays. Analyst.

[42] Marguí E., Zawisza B., Sitko R. (2014). Trace and ultratrace analysis of liquid samples by X-ray fluorescence spectrometry. TrAC, Trends Anal. Chem..

[43] Sardans J., Montes F., Penuelas J. (2011). Electrothermal atomic absorption spectrometry to determine As, Cd, Cr, Cu, Hg, and Pb in soils and sediments: a review and perspectives. Soil Sediment Contam..

[44] Yildiz Z., Arslan G., Tor A. (2011). Preconcentrative separation of chromium (III) species from chromium (VI) by cloud point extraction and determination by flame atomic absorption spectrometry. Microchim. Acta.

[45] Ulusoy H.I., Gürkan R., Yılmaz Ö., Akçay M. (2012). Development of a cloud point extraction and preconcentration method for chromium (III) and total chromium prior to flame atomic absorption spectrometry. J. Anal. Chem..

[46] Saleh S.M., Ali R., Ali I.A. (2017). A novel, highly sensitive, selective, reversible and turn-on chemi-sensor based on Schiff base for rapid detection of Cu (II). Spectrochim. Acta Mol. Biomol. Spectrosc..

[47] Saleh S.M., Ali R., Elshaarawy R.F. (2016). A ratiometric and selective fluorescent chemosensor for Ca (II) ions based on a novel water-soluble ionic Schiff-base. RSC advances.

[48] Saleh S.M., Müller R., Mader H.S., Duerkop A., Wolfbeis O.S. (2010). Novel multicolor fluorescently labeled silica nanoparticles for interface fluorescence resonance energy transfer to and from labeled avidin. Anal. Bioanal. Chem..

[49] Wang X., Wang Q., Chen Y., Li J., Pan R., Cheng X., Ng K.W., Zhu X., He T., Cheng J., Tang Z. (2021). Metal-to-ligand charge transfer chirality-based sensing of mercury ions. Photon. Res..

[50] de Silva A.P. (2011). Luminescent photoinduced electron transfer (PET) molecules for sensing and logic operations. J. Phys. Chem. Lett..

[51] Gao M., Tang B.Z. (2017). Fluorescent sensors based on aggregation-induced emission: recent advances and perspectives. ACS Sens..

[52] Li X., Rajasree S.S., Yu J., Deria P. (2020). The role of photoinduced charge transfer for photocatalysis, photoelectrocatalysis and luminescence sensing in metal-organic frameworks. Dalton Trans..

[53] Chalmardi G.B., Tajbakhsh M., Bekhradnia A., Hosseinzadeh R. (2017). A highly sensitive and selective novel fluorescent chemosensor for detection of Cr^3+^ based on a Schiff base. Inorg. Chim. Acta..

[54] Batista R.M., Costa S.P., Raposo M.M.M. (2013). Naphthyl-imidazo-anthraquinones as novel colorimetric and fluorimetric chemosensors for ion sensing. J. Photochem. Photobiol. Chem..

[55] He W.W., Yang G.S., Tang Y.J., Li S.L., Zhang S.R., Su Z.M., Lan Y.Q. (2015). Phenyl groups result in the highest benzene storage and most efficient desulfurization in a series of isostructural metal–organic frameworks. Chem.--Eur. J..

[56] Ali R., Alminderej F.M., Messaoudi S., Saleh S.M. (2021). Ratiometric ultrasensitive optical chemisensor film based antibiotic drug for Al(III) and Cu(II) detection. Talanta.

[57] Saleem M., Lee K.H. (2015). Optical sensor: a promising strategy for environmental and biomedical monitoring of ionic species. Rsc Advances.

[58] Facchiano A., Ragone R. (2003). Modification of Job's method for determining the stoichiometry of protein-protein complexes. Anal. Biochem..

[59] Chen X.C., Tao T., Wang Y.G., Peng Y.X., Huang W., Qian H.F. (2012). Azo-hydrazone tautomerism observed from UV-vis spectra by pH control and metal-ion complexation for two heterocyclic disperse yellow dyes. Dalton Trans..

[60] Tajbakhsh M., Chalmardi G.B., Bekhradnia A., Hosseinzadeh R., Hasani N., Amiri M.A. (2018). A new fluorene-based Schiff-base as fluorescent chemosensor for selective detection of Cr^3+^ and Al^3+^. Spectrochim. Acta Mol. Biomol. Spectrosc..

[61] Dhineshkumar E., Iyappan M., Anbuselvan C. (2020). A novel dual chemosensor for selective heavy metal ions Al^3+^, Cr^3+^ and its applicable cytotoxic activity, HepG2 living cell images and theoretical studies. J. Mol. Struct..

[62] Tawfik S.M., Farag A.A., Kobisy A.S., Elged A.H., Abusaif M.S., Ammar Y.A., Ragab A. (2023). Blue-green emitting cationic thiazole surfactants-based paper devices for highly sensitive and selective fluorescence detection of chromium oxyanions in wastewater. Microchem. J..

[63] Deshapande N., Belavagi N.S., Panchamukhi S.I., Rabinal M.H., Khazi I.A.M. (2014). Synthesis and optoelectronic properties of thieno [2,3-b] thiophene based bis 1,3,4-oxadiazole derivatives as blue fluorescent material for use in organic light emitting diodes. Opt. Mater..

[64] Elshaarawy R.F., Ali R., Saleh S.M., Janiak C. (2017). A novel water-soluble highly selective “switch-on” ionic liquid-based fluorescent chemi-sensor for Ca (II). J. Mol. Liq..

[65] Zhu W., Yang L., Fang M., Wu Z., Zhang Q., Yin F., Huang Q., Li C. (2015). New carbazole-based Schiff base: colorimetric chemosensor for Fe^3+^ and fluorescent turn-on chemosensor for Fe^3+^ and Cr^3+^. J. Lumin..

[66] Kolcu F., Erdener D., Kaya İ. (2020). A Schiff base based on triphenylamine and thiophene moieties as a fluorescent sensor for Cr (III) ions: synthesis, characterization and fluorescent applications. Inorg. Chim. Acta..

[67] Guha S., Lohar S., Banerjee A., Sahana A., Hauli I., Mukherjee S.K., Matalobos J.S., Das D. (2012). Thiophene anchored coumarin derivative as a turn-on fluorescent probe for Cr^3+^: cell imaging and speciation studies. Talanta.

[68] Saluja P., Sharma H., Kaur N., Singh N., Jang D.O. (2012). Benzimidazole-based imine-linked chemosensor: chromogenic sensor for Mg^2+^ and fluorescent sensor for Cr^3+^. Tetrahedron.

[69] Kumawat L.K., Mergu N., Asif M., Gupta V.K. (2016). Novel synthesized antipyrine derivative based “Naked eye” colorimetric chemosensors for Al^3+^ and Cr^3+^. Sensor. Actuator. B Chem..

[70] Jang Y.J., Yeon Y.H., Yang H.Y., Noh J.Y., Hwang I.H., Kim C. (2013). A colorimetric and fluorescent chemosensor for selective detection of Cr^3+^ and Al^3+^. Inorg. Chem. Commun..

[71] Chalmardi G.B., Tajbakhsh M., Bekhradnia A., Hosseinzadeh R. (2017). A highly sensitive and selective novel fluorescent chemosensor for detection of Cr^3+^ based on a Schiff base. Inorg. Chim. Acta..

