# Supplementary material for: A novel organic chromo-fluorogenic optical sensor for detecting chromium ions
Source: Heliyon. 2024 Sep 7;10(17):e37480. doi: 10.1016/j.heliyon.2024.e37480 (PMC11413675; doi:10.1016/j.heliyon.2024.e37480)
Supplement: Multimedia component 1 [file mmc1.docx]

**Supplementary Information**

**Title: A novel organic chromo-fluorogenic optical sensor for detecting chromium ions.**

**Sayed M. Saleh ^a^*, Reham Ali ^a^, Azizah Algreiby^a^, Bayader Alfeneekh^a^, Ibrahim A.I. Ali ^b^**

^a^Department of Chemistry, College of Science, Qassim University, Buraidah 51452, Saudi Arabia

^b^Chemistry Department, Faculty of Science, Suez Canal University, Ismailia, Egypt

*** Corresponding Author:**

***E-mail address:*** E.Saleh@qu.edu.sa

| 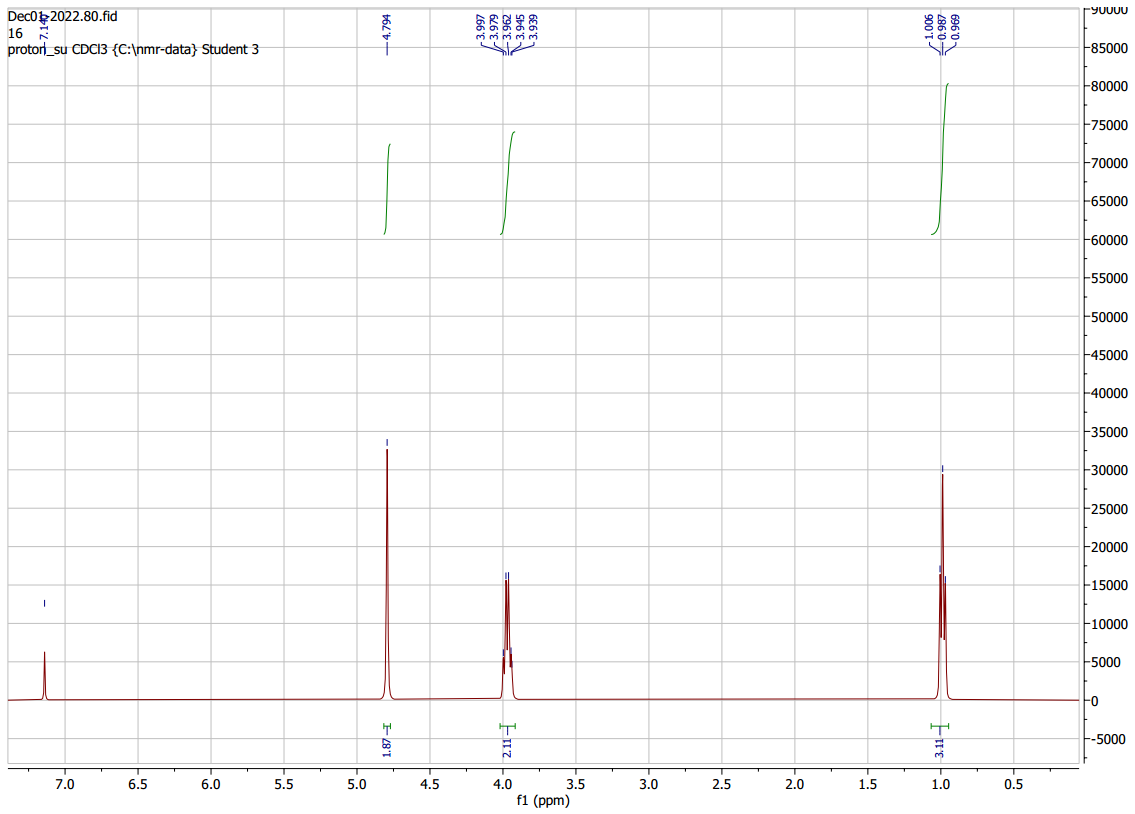 |
| --- |
| **Fig. S1**. ^1^H NMR Spectra of the 3D compound. |

| 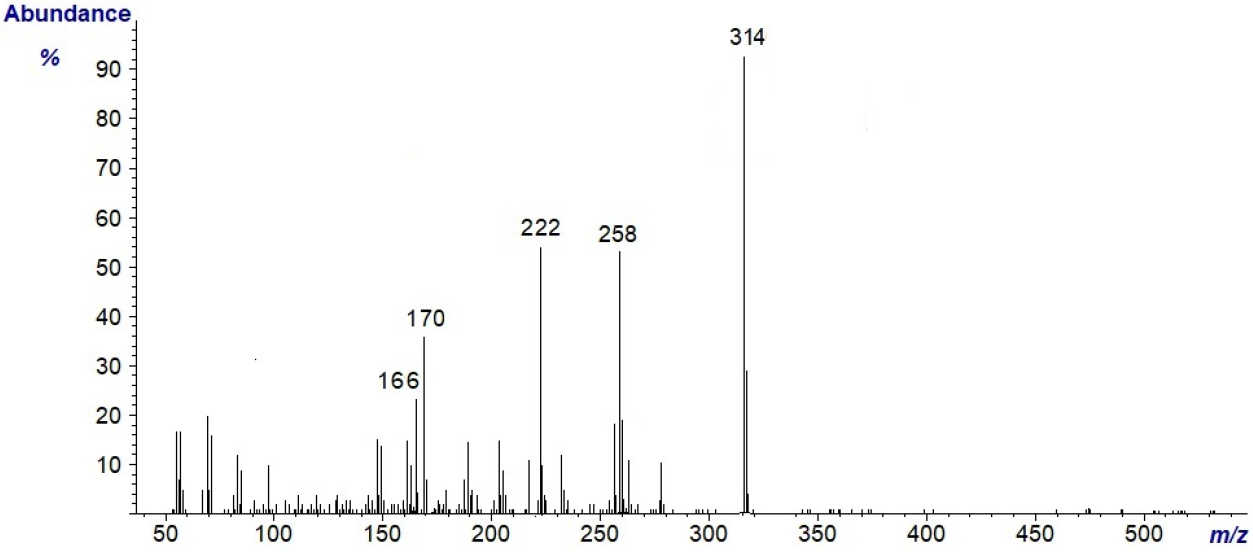 |
| --- |
| **Fig. S2**. Mass spectra of the 3D compound. |
